# Decisions regarding admission to the ICU and international initiatives to improve the decision-making process

**DOI:** 10.1186/s13054-017-1749-3

**Published:** 2017-07-04

**Authors:** Christopher Bassford

**Affiliations:** 1 General Critical Care, University Hospital, Clifford Bridge Road, Coventry, CV2 2DX UK; 2 University of Warwick, Coventry, UK

**Keywords:** Intensive care triage, Decision-making, Intensive care resource allocation, Intensive care unit admissions

Whether a critically ill patient should or should not be offered life-supporting treatment in the intensive care unit (ICU) is arguably the most important decision that is regularly made on behalf of a patient; deciding not to admit somebody may mean that their death is inevitable. Yet these decisions are often made in the face of uncertain information, time constraints and without the patient being able to participate in discussions. In this context it is perhaps surprising that more research and guidance has not addressed this area of practice.

There are three overarching considerations that must influence the decision not to admit a patient to ICU. Firstly, that an individual patient’s preference may be to decline intensive care treatments. Secondly, that the burden of invasive and distressing treatments on the ICU may outweigh any potential benefit. Thirdly, that the ICU is a labour and resource-intensive endeavour: resources are not available for every patient to be admitted. Intensivists are provided with a limited amount of resources and are, usually implicitly, charged with doing as much good as possible with what is available. This means that at times of high demand or limited resource availability, patients who have less clear-cut potential to benefit from the ICU may not be admitted [1, 2]. Managing resources is an unavoidable part of the intensivist’s job.

In this issue Nicolas Chin-Yee and colleagues shine more light on the interaction of these three issues by studying the impact of admission to the ICU for older patients. Using retrospective financial and medical record data, they have studied resource implications, outcomes and patient preferences. For this cohort of patients, treatment on the ICU is not only expensive, but its outcomes are poor. Furthermore, a fifth of patients may have preferred treatment focused on their comfort rather than life-supporting measures [3]. Their findings support the assertion that if decision-making surrounding admission to the ICU could be improved, this would have objective benefits. It could save many patients from being subjected to treatments that do not help them, and could free available resources for patients who can both benefit from them and who would opt for this type of treatment.

How, then, to improve decision-making surrounding admission to the ICU and achieve these objective benefits? The first step, as in any other field of medical practice, is to understand and articulate effectively the processes and factors involved. Improving the nomenclature surrounding this process is an important first step. In the USA in 2016, the Society of Critical Care Medicine (SCCM) published guidance to define what might be regarded as “futile” or “inappropriate” treatment [4]. These definitions help to focus discussion and clarify thinking around these issues.

Secondly, we need the right information to inform our decisions. Initiatives such as the Eldicus project, which has developed statistics-based triage tools for patients referred to the ICU [5], may help: but statistical tools and quantitative outcome data alone cannot determine what is right for any individual. Outcomes relevant to each patient, their values and wishes must be taken into account. This can be a challenge when a patient cannot communicate effectively. In the UK, the traditional “Do not attempt resuscitation” charts are being replaced by “Recommended Summary Plan for Emergency Care and Treatment” (ReSPECT) forms: these give patients a chance to record what values and outcomes are important to them so that health care professionals can take this into consideration when planning emergency treatment such as intensive care [6].

Thirdly we must educate ourselves and design systems for best practice in decision-making. In 2016 a working party of the World Federation of Societies of Intensive and Critical Care Medicine produced guidance on triaging patients to intensive care. They brought together evidence and expert opinion to address four important questions: who will benefit from intensive care; who makes the decision whether a patient should be admitted to intensive care; what in-hospital factors limit the ability to admit a patient to intensive care; and what other factors should influence whether or not a patient should be admitted to the ICU [7]? In the USA, the SCCM has also produced guidance on how institutions might establish criteria for admission and triage [8]. These are undoubtedly valuable documents, and should be built upon to provide guidance for individual clinicians at the bedside. Standards and education in decision-making practice will protect and guide patients and clinicians when faced with these difficult clinical and ethical challenges.

Intensive care resource provision will never be sufficient without clear and rational decision-making regarding admission to ICU: the SCCM guidelines referred to originate from a nation which is able to spend between $121 and $263 billion on critical care [9]. Indeed, merely providing more resources may result in more waste and more harm to patients. In 2015 a study compared outcomes between units with high bed availability and low bed availability. The findings were that more beds may mean the admission of patients who are less likely to benefit from the ICU either because they are too well or too sick to benefit [10]. Our approach therefore must be different. We must develop standards and guidelines for decision-making surrounding intensive care admission as in any other part of our practice. Decisions should be based on the best evidence, with clear reasoning, communication and review. We must educate future generations of intensivists so that they are better equipped to make such decisions, and we must design the systems to support high-quality decision-making practice. If we can do this while keeping the patient at the heart of our decision-making, and being clear as we articulate the rationale for each decision, then, as Chin-Yee and colleagues suggest, it may result in the objective benefits we seek.

## Abbreviations

FICM: Faculty of Intensive Care Medicine
ICU: intensive care unit
NIHR: National institute for health research
ReSPECT: Recommended Summary Plan for Emergency Care and Treatment
SCCM: Society of Critical Care Medicine
UK: United Kingdom
USA: United States of America

## References

[1] Garrouste-Orgeas M, Tabah A, Vesin A, Philippart F, Kpodji A, Bruel C, Gregoire C, Max A, Timsit JF, Misset B (2013). The ETHICA study (part II): simulation study of determinants and variability of ICU physician decisions in patients aged 80 or over. Intensive Care Med.

[2] Stelfox HT, Hemmelgarn BR, Bagshaw SM, Gao S, Doig CJ, Nijssen-Jordan C, Manns B (2012). Intensive care unit bed availability and outcomes for hospitalized patients with sudden clinical deterioration. Arch Intern Med.

[3] Chin-Yee NJ, D'Egido G, Thavorn K, Heyland D, Kyeremanteng K. Cost analysis of the very elderly admitted to intensive care unit. Crit Care. 2017;21(1):109. 10.1186/s13054-017-1689-y PMC5433056 28506243

[4] Kon AA, Shepard EK, Sederstrom NO, Swoboda SM, Marshall MF, Birriel B, Rincon F (2016). Defining futile and potentially inappropriate interventions: a policy statement from the Society of Critical Care Medicine Ethics Committee. Crit Care Med.

[5] Sprung CL, Baras M, Iapichino G, Kesecioglu J, Lippert A, Hargreaves C, Pezzi A, Pirracchio R, Edbrooke DL, Pesenti A (2012). The Eldicus prospective, observational study of triage decision making in European intensive care units: part I—European Intensive Care Admission Triage Scores. Crit Care Med.

[6] Fritz Z, Slowther AM, Perkins GD (2017). Resuscitation policy should focus on the patient, not the decision. BMJ.

[7] Blanch L, Abillama FF, Amin P, Christian M, Joynt GM, Myburgh J, Nates JL, Pelosi P, Sprung C, Topeli A (2016). Triage decisions for ICU admission: report from the Task Force of the World Federation of Societies of Intensive and Critical Care Medicine. J Crit Care.

[8] Nates JL, Nunnally M, Kleinpell R, Blosser S, Goldner J, Birriel B, Fowler CS, Byrum D, Miles WS, Bailey H (2016). ICU Admission, discharge, and triage guidelines: a framework to enhance clinical operations, development of institutional policies, and further research. Crit Care Med.

[9] Coopersmith CM, Wunsch H, Fink MP, Linde-Zwirble WT, Olsen KM, Sommers MS, Anand KJ, Tchorz KM, Angus DC, Deutschman CS (2012). A comparison of critical care research funding and the financial burden of critical illness in the United States. Crit Care Med.

[10] Robert R, Coudroy R, Ragot S, Lesieur O, Runge I, Souday V, Desachy A, Gouello JP, Hira M, Hamrouni M (2015). Influence of ICU-bed availability on ICU admission decisions. Ann Intensive Care.

[11] Bassford CR, Slowther A-M, Rees K, Krucien N, Fritz Z, Quinton S, White C, Symons S, Perkins GD, Griffiths F, Dale J, Ryan M. Gatekeeping in intensive care: understanding and improving the decision-making process surrounding admission to the intensive care unit. University of Warwick, Coventry, UK; 2015. http://www.journalslibrary.nihr.ac.uk/programmes/hta/131014. Accessed May 2017.

